# COMPARING SELF-REPORTED MEASURES OF SUBJECTIVE COGNITIVE DECLINE

**DOI:** 10.1093/geroni/igad104.1892

**Published:** 2023-12-21

**Authors:** Benjamin Olivari, John Omura, John Shean, Lisa McGuire

**Affiliations:** CDC, Atlanta, Georgia, United States; Centers for Disease Control and Prevention, Atlanta, Georgia, United States; Alzheimer’s Association, Chicago, Illinois, United States; CDC, Atlanta, Georgia, United States

## Abstract

Symptoms of subjective cognitive decline (SCD) can be early indicators of possible future dementia. Estimates of self-reported SCD prevalence are available from various data sources including the Behavioral Risk Factor Surveillance System’s (BRFSS) current Cognitive Decline Optional Module. Recent revisions to the Module were proposed based on input from subject matter experts, including reducing introductory phrasing and refining question and response options. It is unknown how these changes may impact estimates of SCD prevalence. To assess whether prevalence of SCD differs based on Module phrasing and conceptualizations of SCD-related questions, this analysis used data (n=3526) from the 2022 FallStyles survey. Respondents were randomly assigned to one of two groups receiving different sets of Module questions: group A received questions mirroring the former language and group B received the revised phrasing. Estimates were weighted to match the 2021 US Current Population Survey. SCD prevalence among respondents aged ≥18 years was 8.9% in group A versus 16.9% in group B. Among adults aged ≥45 years, SCD prevalence in group A (9.3%) was similar to the 2019-2020 BRFSS estimate (9.8%), while it was nearly double (17.6%) in group B. Within both groups, SCD prevalence was higher among those earning less than $25,000, with lower educational attainment, and who identified as female than their counterparts. Simplified prompts and refined questions may provide higher estimates of SCD prevalence. Given the subjective interpretation of respondents’ own memory changes involved in measuring SCD, understanding the impact of question phrasing and language is critical when interpreting resulting data.

